# TLR4 Enhances Cerebral Ischemia/Reperfusion Injury via Regulating NLRP3 Inflammasome and Autophagy

**DOI:** 10.1155/2023/9335166

**Published:** 2023-02-25

**Authors:** Li Mao, Da-Hua Wu, Guo-Huang Hu, Jian-Hu Fan

**Affiliations:** ^1^Department of Basic Medicine, Changsha Health Vocational College, Changsha, 410600 Hunan, China; ^2^Department of Neurology, Affiliated Hospital of Hunan Academy of Traditional Chinese Medicine, Changsha, 410006 Hunan, China; ^3^The Affiliated Changsha Hospital of Hunan Normal University, Changsha, 410006 Hunan, China

## Abstract

Ischemic stroke is a kind of central nervous disease characterized by high morbidity, high mortality, and high disability. Inflammation and autophagy play important roles in cerebral ischemia/reperfusion (CI/R) injury. The present study characterizes the effects of TLR4 activation on inflammation and autophagy in CI/R injury. An in vivo CI/R rat injury model and an in vitro hypoxia/reoxygenation (H/R) SH-SY5Y cell model were established. Brain infarction size, neurological function, cell apoptosis, inflammatory mediators' levels, and gene expression were measured. Infarction, neurological dysfunction, and neural cell apoptosis were induced in CI/R rats or in H/R-induced cells. The expression levels of NLRP3, TLR4, LC3, TNF-*α*, interleukin-1 (IL-1), interleukin-6 (IL-6), and interleukin-18 (IL-18) clearly increased in I/R rats or in H/R-induced cells, while TLR4 knockdown significantly suppressed NLRP3, TLR4, LC3, TNF-*α*, and interleukin-1/6/18 (IL-1/6/18) in H/R-induced cells, as well as cell apoptosis. These data indicate that TLR4 upregulation induced CI/R injury via stimulating NLRP3 inflammasome and autophagy. Therefore, TLR4, is a potential therapeutic target to improve management of ischemic stroke.

## 1. Introduction

Ischemic stroke (IS) is a common disease in the clinic, which has high morbidity, high mortality, and high disability. It imposes heavy economic and emotional burdens on society [1]. Ischemia is an aging-related disease that results from cardiac insufficiency and brain vascular occlusion. Its occurrence increases with age and is aggravated by a sedentary lifestyle. Circulatory dysfunction causes both glucose and oxygen insufficiencies which are the main causes of ischemic stroke [2, 3] of the blood supplied by either thrombolysis or thrombectomy which is the main therapeutic regimen for reversal of the declines in glucose and oxygenation. However, blood flow restoration will rapidly result in cerebral ischemia/reperfusion (CI/R) injury [4]. Recently, studies have demonstrated that during the process of CI/R injury, the insulted brain tissues undergo severe oxidative stress, excitotoxicity, inflammatory reaction, autophagy, and edema [2, 3, 5]. Among these responses, inflammation and autophagy have received extensive attention, but the cellular mechanisms are unclear that mediate these responses to CI/R injury. Therefore, improved therapeutic management of ischemic stroke awaits clarification of the mechanisms that mediate CI/R [6].

Inflammation and autophagy are key players in mediating the neurological deficits that develop following a CI/R accident [6]. During the process of ischemia-reperfusion, inflammation develops in the brain tissue due to increases in infiltration of inflammatory cells such as macrophages and microglia, and they release many proinflammatory factors, such as TNF-*α* and interleukin-1*β*/18 (IL-1*β*/18), ultimately inducing brain tissue damage [7–9]. This inflammatory process is mediated through the formation and activation of NLRP3 inflammasomes in MCAO (middle cerebral artery occlusion) mouse models [9]. Evidence for their activation stems from NLRP3-induced rises in IL-1*β* release [9]. In addition, it was shown that crosstalk exists between the mediators controlling NLRP3 inflammasome formation and autophagy in CI/R injury [6]. Autophagy activation coexists with NLRP3 inflammasome induction in neuronal apoptosis or death in different models of CI/R injury (global ischemia, ischemia, or hypoxic ischemia). However, the molecular mechanisms are unclear that underlie these pathological responses and they need clarification.

Recent studies showed that Toll-like receptor 4 (TLR4) activation in stress-induced hypoxia of neuronal cells induces an inflammatory response. TLR4 involvement is evident since they undergo upregulation during an ischemic insult, whereas this response is suppressed in TLR4-deficient/knockout mice [10, 11]. These effects suggest that CI/R injury-induced TLR4 upregulation is an inadvertent injurious side effect to such stress. However, it is still unclear whether TLR4 expression and activation directly contribute to the side effects of Cl/R-induced injury through upregulating and stimulating NLRP3 inflammasomes and autophagy in brain tissues.

We show here that hypoxia-induced TLR4 upregulation and activation during reperfusion contribute to mediating through NLRP3 inflammasomes which increase in inflammation and autophagy in brain neuronal tissue in vivo and in vitro. These results provide novel insight into the pathogenesis and treatment of ischemic stroke.

## 2. Materials and Methods

### 2.1. Animal Experiments

SD male rats (*n* = 16; 8 weeks old, 250 g in weight) were provided by Hunan SJA Laboratory Animal Co., Ltd. The rats were maintained at 25°C, 65% humidity on a cycle of 12 h light/12 h dark. This investigation was approved by the Ethics Committee of the Affiliated Changsha Hospital of Hunan Normal University, and the performed experiments comply with the guidelines of National Institutes of Health (NIH).

A SD rat CI/R model was created following the method established by Mao et al. [12] or Zuo et al. [4]. The rats were lightly anesthetized by intraperitoneal injection with pentobarbital sodium. The occlusion of rat's anterior cerebral artery was performed using a nylon monofilament. After 2 h of occlusion, the rats underwent 24 h of reperfusion. The sham group only received a skin incision without internal carotid artery occlusion. Neurological function was assessed following the 24 h reperfusion, and brain tissue was sampled for TTC (2,3,5-tripenyltetrazolium chloride) staining or other relevant analysis, such as gene expression.

### 2.2. Cell Culture

The SH-SY5Y cell line was obtained from the CTCC of Chinese Academy of Sciences and authenticated using STR (short tandem repeat) profiling. Cells were cultured at 37°C, 95% air, and 5% CO_2_ in a compound medium containing DMEM, 10% fetal bovine serum, and antibiotics.

### 2.3. In Vitro Model In Vitro Model of Ischemic Stroke (IS)

To mimic ischemic stroke in vitro, an H/R (hypoxia/reoxygenation) model was used. SH-SY5Y cells were subjected to 4 h of hypoxia followed by 20 h reoxygenation. During hypoxia, the SH-SY5Y cells were only induced using DPBS (Dulbecco's phosphate-buffered saline), while during the reoxygenation, the DPBS was replaced with normal medium (DMEM medium containing 10% FBS).

### 2.4. Neurological Function Evaluation

The neurological function assessment followed a 5-point rating scale [4]. According to the rating scale, 0 means normal, 1 means the left forepaw cannot be straightened, 2 represents a decrease in the grip strength of the forepaw, 3 means turning to the left when pulling the tail, and 4 means spontaneous rotation.

### 2.5. Infarct Size Measurement

TTC (2,3,5-tripenyltetrazolium chloride) staining was performed for brain infarction size measurement following previous described methods [4]. Briefly, brain sections (0.2-0.3 cm in thickness) were incubated with 2% TTC solution for half an hour at 25°C in the dark and then imaged. The total infarction size (cm^3^) of sections was calculated by the equation: total infarction size = infarction area (cm^2^) of each section^∗^ section thickness.

### 2.6. Cell Apoptosis Characterization

TUNEL/Hoechst double-labelling was used for assaying the apoptosis of brain tissues [13]. Brain sections were handled as follows: immersed in 4% w/v paraformaldehyde (25°C, 10 min) for fixing, immersed in paraformaldehyde plus acetic acid (4°C, 5 min) for postfixing, and immersed in reaction solutions containing equilibration buffer and working strength deoxynucleotide transferase (TdT) (35°C, 1 h) and blotting off, followed by incubation with Hoechst 33342 (25°C, 5 min). At the end, images were captured using epifluorescence microscopy (×200 amplifications), and the number of TUNEL-positive cells was counted.

The cell apoptosis analysis was performed as previously described [4]. SH-SY5Y cell suspension (100 cells/ml) was mixed with FITC-conjugated Annexin V and propidium iodide and maintained in the dark at 25°C for 15 min. Subsequently, the cells apoptosis was detected using a flow cytometer.

### 2.7. Gene Expression Knockdown

Loss of TLR4 function was obtained with the following transfection procedure in SH-SY5Y cells. Mixtures were prepared by mixing siRNA having the TLR4 (R: UGUUCUAGAAUUAAUAAGCCC sequence directed against R: GCUUAUUAAUUCUAGAACAAA) and Lipofectamine 2000. SH-SY5Y cells were transfected for 24 h.

### 2.8. Determination of Inflammatory Mediators

An ELISA kit was used for determining the levels of TNF-*α* (Beyotime), L-1*β* (Beyotime), IL-6 (Beyotime), or IL-18(Beyotime) according to the manufacturer instructions. Briefly, SH-SY5Y cell supernatants were collected, and the optical absorbance at 540 nm was determined using a microplate reader.

### 2.9. Real-Time PCR

Real-time PCR was conducted following described methods [4]. Briefly, total RNA extracted using a TRizol Reagent kit (TakaRa) both its purity and concentration was determined spectrophotometrically. A reverse transcription system and a real-time PCR reaction system were established to quantitatively determine the expression levels of TLR4, NLRP3, and LC3 according to the instructions of a transcription kit (DRR037A; TaKaRa) and a SYBR Premix Ex Taq kit (TaKaRa). The amplification reaction was conducted using the ABI 7300 Real-Time PCR System. PCR primers used as follows: TLR4 (F: 5′-CCGCTCTGGCATCATCTTCA-3′; R: 5′-TCCCACTCGAGGTAGGTGTT-3′), NLRP3 (F: 5′-CTGCAGAGCCTACAGTTGGG-3′; R: 5′-GTCCTGCTTCCACACCTACC-3′), and *β*-actin (F: 5′-CCCATCTATGAGGGTTACGC-3′; R: 5′-TTTAATGTCACGCACGATTTC-3′).

### 2.10. Western Blotting

Total protein was extracted using an extracting solution containing 1% PMSF, and its concentration was determined. SDS-PAGE (on 10% gel) was used for target protein isolation, and they were electrotransfered to PVDF membranes. Membranes were incubated with primary antibodies (4°C, 16 h) and secondary antibodies (25°C, 2 h) successively. The intensity of band signals was detected using enhanced chemiluminescence and ChemiDoc XRS System (Bio-Rad). The primary antibodies include rabbit anti-TLR4, -NLRP3, LC3-I, and LC3-II. *β*-Actin was used to establish protein loading equivalence. All primary antibodies were provided by Santa Cruz.

### 2.11. Statistical Analysis

SPSS 21 was used for statistical analysis. Data are presented as mean ± SD. One-way analysis of variance and independent sample *t*-test were used to calculate differences between groups. *P* values <0.05 were considered significant.

## 3. Results

### 3.1. Severe Damage Occurs in CI/R Injury Rat

**Figure 1:**
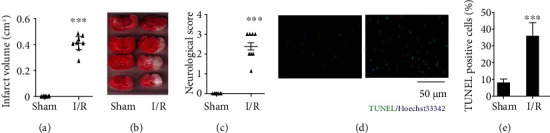
Neuronal histological changes induced in CI/R injury rat. (a) Infarction size. (b) Representative images of TTC staining. (c) Neurological function analysis. (d) Representative images of TUNEL/Hoechst double staining. (e) TUNEL-positive cell numbers. The data expressed as *mean* ± *SD* (*n* = 8). ^∗∗∗^*P* < 0.001 vs. sham.

Figures 1(a) and 1(b) compare the effects of exposure to the I/R stress on neuronal histology with that in the sham group. The results clearly show that imposing the I/R stress induced a brain infarct, whereas the sham group histology was unchanged.  Figure 1(c) shows the results of the neurological function assay, and they indicate that in the I/R group, the I/R stress induced neurological dysfunction (Figure 1(c)). Figures 1(d) and 1(e) show that the brain tissues in the I/R rats had more TUNEL-positive cells, which suggest that they underwent apoptosis. These data confirm that that this I/R regimen induced severe neuronal structural and functional damage.

### 3.2. CI/R Injury Upregulates Proinflammatory Cytokine Release

**Figure 2:**
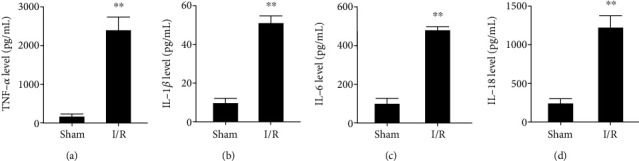
CI/R injury enhances inflammatory mediator release. (a) TNF-*α* level. (b) IL-1*β* level. (c) IL-6 level. (d) IL-18 level. The data expressed as *mean* ± *SD*. ^∗∗^*P* < 0.01 vs. sham.

Since inflammation is the leading cause of CI/R injury, we determined if increases in proinflammatory mediator release accompanies this response. The results in Figures 2(a)–2(d) show that the levels of TNF-*α* and interleukin-1*β*/6/18 (1L-1*β*/6/18) significantly increased in the I/R rat group compared to their corresponding levels in the sham group.

### 3.3. CI/R Injury Upregulates NLRP3, TLR4, and LC3 Expression Levels

**Figure 3:**
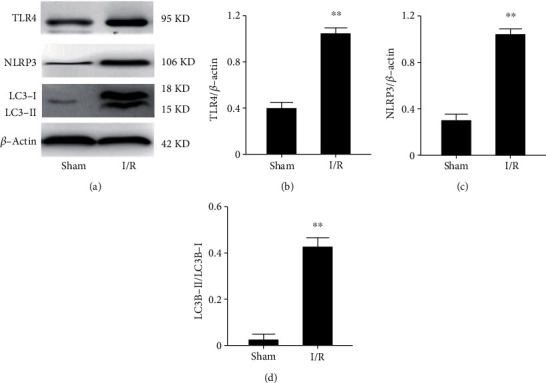
CI/R injury increases the expression of NLRP3, TLR4, and LC3. (a) Protein expression level. (b) The ratio of TLR4 to *β*-actin. (c) The ratio of NLRP3 to *β*-actin. (d) The ratio of LC3-I to LC3-II. The data expressed as *mean* ± *SD*. ^∗∗^*P* < 0.01 vs. control vs. sham.

The effects were determined of CI/R injury on relevant inflammation gene expression levels. The results shown in Figures 3(a) and 3(b) indicate that such stress increased the TLR4 expression level relative to its sustained level in the sham group. Consistently, the NLRP3 gene expression level also underwent upregulation in the I/R stressed rats (c.f. Figures 3(a) and 3(c)). This agreement suggested that TLR4 and NLRP3 upregulation are closely associated with the inflammation response in the I/R rat. Considering that there is an interplay between inflammation and autophagy, we then measured the expression level of LC3 (a biomarker of apoptosis) [6]. The results shown in Figures 3(a) and 3(d) clearly show that the ratio of LC3-II to LC3-I increased in the I/R rats. This correspondence between the increases in the LC3-II/LC3-1(biomarker of apoptosis) [6] ratio and NLRP3 inflammasome upregulation suggests that these responses are linked to one another in CI/R injury.

### 3.4. Correspondence between the Effects of TLR4 Knockdown on NLRP3 and LC3 in H/R-Induced SH-SY5Y Cells and Those in the CI/R Rat Group

**Figure 4:**
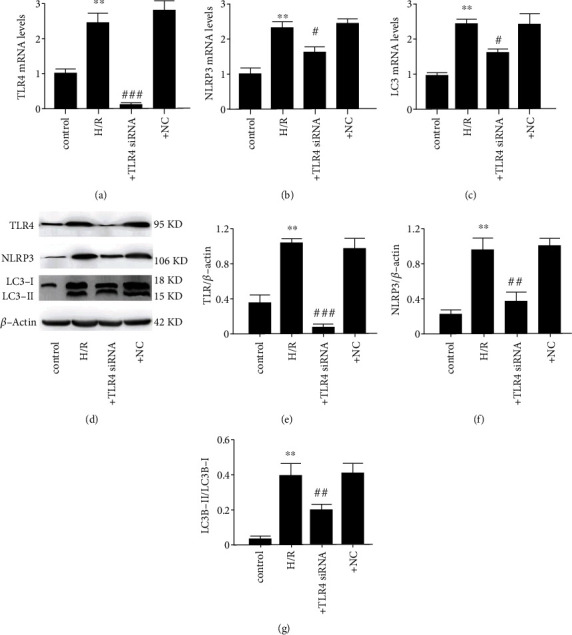
TLR4 knockdown suppresses rises in NLRP3 and LC3 in H/R-induced SH-SY5Y cells. (a) TLR4 mRNA level. (b) NLRP3 mRNA level. (c) LC3 mRNA level. (d) protein expression level. (e) The ratio of TLR4 to *β*-actin. (f) The ratio of NLRP3 to *β*-actin. (g) The ratio of LC3-I to LC3-II. The data expressed as *mean* ± *SD*. +TLR4 siRNA: cells treated with siRNA against TLR4 (100 nm); +NC siRNA: cells with negative control siRNA (100 nm). ^∗∗^*P* < 0.05 vs. control; ^#^*P* < 0.05 vs. H/R; ^##^*P* < 0.01 vs. H/R; ^###^*P* < 0.001 vs. H/R.

We determined if there is a correspondence between in vivo H/R-induced upregulation of TLR4, NLRP3, and LC3 expression levels in vivo and those induced by exposing SH-SY5Y cells to this stress (Figures 4(a)–4(g)). The results shown in Figures 4(a)–4(g) confirm that H/R exposure under both conditions upregulates these mediators through stimulating TLR4 activity. The results in Figure 4(a) show that transfection with the TLR4 gene silencing siRNA significantly inhibited the expression of TLR4 in SH-SY5Y cells that underwent H/R, but the NC physiologically irrelevant siRNA had no effect. Figures 4(d) and 4(e) show that transfection of this TLR4 siRNA inhibited the TLR4 protein expression level in H/R-induced SH-SY5Y cells. In addition, TLR4 knockdown inhibited the expression levels of NLRP3 and LC3 in H/R-induced SH-SY5Y cells, while the NC siRNA had no inhibitory effect on any of their expression levels (c.f. Figures 4(b)–4(d), 4(f), and 4(g)). These data confirm that H/R upregulates NLRP3 inflammasome and autophagy through increases in TLR4 activity.

### 3.5. TLR4 Knockdown Suppresses Inflammatory Mediator Release in H/R-Induced SH-SY5Y Cells

**Figure 5:**
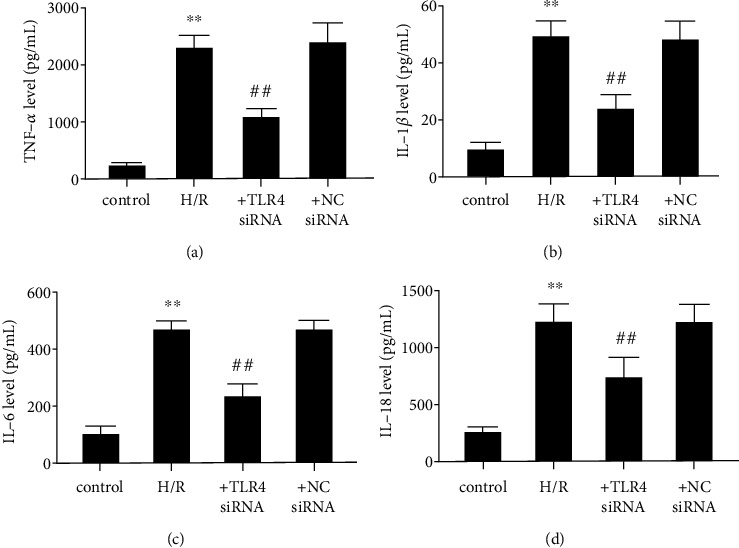
TLR4 knockdown inhibits inflammatory mediator release in H/R treated SH-SY5Y cells. (a) TNF-*α* level. (b) IL-1*β* level. (c) IL-6 level. (d) IL-18 level. The data expressed as *mean* ± *SD*. ^∗∗^*P* < 0.01 vs. control; ^##^*P* < 0.01 vs. H/R.

To determine if exposure of SH-SY5Y cells to the H/R stress mediates increases in proinflammatory cytokine release through stimulating the TLR4 receptor, the effects of TLR4 knockdown were evaluated on these rises in H/R stressed SH-SY5Y cells. Consistent with the animal results, the release of TNF-*α* and interleukin -1*β*/6/18 (1L-1*β*/6/18) significantly increased in H/R-induced SH-SY5Y cells, while TLR4 knockdown obviously reduced their release (Figures 5(a)–5(d)). These results confirm TLR4 involvement in mediating H/R-induced increases on proinflammatory cytokine expression levels underlying inflammation in neurons.

### 3.6. TLR4 Knockdown Suppresses H/R-Induced SH-SY5Y Apoptosis

**Figure 6:**
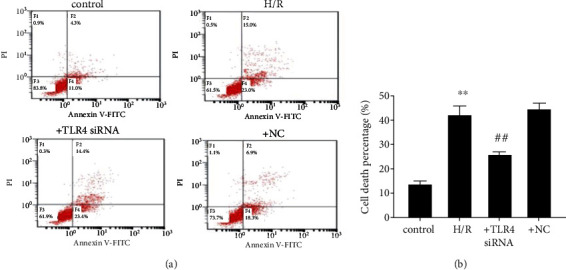
(a) TLR4 knockdown reduces increases in cell apoptosis in H/R-induced SH-SY5Y cells. Representative images of flow cytometry. (b) Cell death percentage. The data expressed as *mean* ± *SD*. ^∗∗^*P* < 0.01 vs. control; ^##^*P* < 0.01 vs. H/R.

The association was determined between H/R-induced TLR4 activation and apoptosis in SH-SY5Y cells. The results shown in Figures 6(a) and 6(b) indicate that H/R treatment obviously induced increases in SH-SY5Y while TLR4 knockdown obviously inhibited this response. Therefore, H/R induces neuronal apoptosis through upregulating and stimulating TLR4 expression levels.

## 4. Discussion

Inflammation exerts key roles in CI/R injury. The present study revealed that the high expression of TLR4 led to CI/R injury via regulating NLRP3 and LC3 upregulation and stimulation. The results indicate that the expression level of TLR4 positively correlates with the expression level of NLRP3 and LC3. The cell apoptosis and the release of inflammatory mediators rose in brain tissues subjected to CI/R or in SH-SY5Y cells. TLR4 knockdown significantly suppressed the expression of TLR4, NLRP3, and LC3 and inhibited apoptosis of SH-SY5Y cells that underwent H/R. These results indicate that TLR4 induces CI/R injury via regulating NLRP3 inflammasome and autophagy. These findings suggest that NLRP3 inhibition is a potential target for ischemic stroke treatment.

Inflammation is activated in response to ischemia/reperfusion in brain tissues. This effect is due to increases in inflammatory cells (macrophages) and the microglia infiltration which release many proinflammatory factors, such as TNF-*α* and interleukin -1*β*/18(1 L-1*β*/18) [14]. During this process, the formation and activation of the NLRP3 inflammasome are critical to the initiation of the inflammatory response. Accordingly, the role of NLRP3 inflammasome in CI/R injury is very widely studied [7, 8]. For example, NLRP3 upregulation was found to be upregulated in MCAO mouse models as well as Bruton's Ttrosine kinase (BTK) which induces responses through regulating the expression level of NLRP3 [9, 15]. Increases in the release of IL-1*β* are a marker of NLRP3 inflammasome activation, which was identified in the brain tissues of MCAO mouse [9]. We found that CI/R- and H/R-induced neuronal injury in vitro and in vivo is associated with increases in the release of TNF-*α* and interleukin-1*β*/18 (1L-1*β*/18), which confirms that NLRP3 activation contributes to mediating these effects. This mimicry confirms the relevance of TLR4-induced increases in inflammation in which H/R and cerebral artery occlusion both upregulated TNF-*α* and interleukin-1*β*/18 (1L-1*β*/18) [9] through NLRP3 inflammasome upregulation and activation. In addition, He et al. recently found that resveratrol can reduce cerebral infarction area and improve neurobiological function of rats that underwent CI/R injury, and its mechanism involves inhibition of NLRP3 inflammasome [16]. These data provide additional proof that exposure to CI/R induces neuronal injury through NLRP3 inflammasome activation. Thus, clarifying the mechanisms that underlie the control of NLRP3 function is very relevant for identifying novel therapeutic approaches to lessen CI/R injury.

Autophagy is a conserved, controlled, and lysosomal-dependent biological process formed during the evolution of eukaryotes and participates in the pathogenesis of many diseases in mammals, such as CI/R injury [7, 17]. In the process of CI/R injury, it was found that autophagy may be a double-edged sword (beneficial or harmful), such as in the ischemic preconditioning model. In this condition, autophagy activation is neuroprotective, while in the CI/R injury model (complete cerebral ischemia and ischemia or hypoxia ischemia model), autophagy activation is instead associated with nerve cell apoptosis or death [18–20]. This suggested that the mechanism of autophagy in CI/R injury is very complex. Therefore, clarifying the mechanism of autophagy in CI/R injury is critical for ischemic stroke therapy, and there is an urgent need warranting extensive basic or clinical research to solve this complex question. Previous studies showed that autophagy accompanies infarction and apoptosis in neuronal brain tissues of CI/R injury rats, while inhibition of autophagy provides protection against neuronal brain damage [20, 21]. For example, Zhang et al. found that deltonin can inhibit autophagy to alleviate cerebral ischemia-reperfusion injury [21]. Consistently, we found that LC3 was obviously upregulated in CI/R injury rats. There was an association between increases in the expression level of LC3 (a biomarker of autophagy) [18] and brain infarction size and TUNEL-positive cell number. These results suggest that there is an association between CI/R injury and autophagy.

It is apparent that the mechanism of autophagy in CI/R injury is very complex, which is mediated by signaling pathways, such as AMPK-mTOR pathway, PI3K/Akt/mTOR pathway, and TLR4/p38/MAPK [19, 21–23]. Among them, the PI3K/Akt/mTOR pathway and the TLR4/P38/MAPK pathway-mediated apoptosis of neurons is recently the focus of numerous reports. Many studies have described that CI/R injury is associated with a disrupted TLR4/P38/MAPK signaling pathway which suggests it is an important target for the treatment of CI/R injury. For example, Huang et al. found that curcumin can alleviate CI/R injury through inhibition of the TLR4/P38/MAPK signaling pathway [24]. Consistently, we found that there is a positive correlation between TLR4 expression levels and autophagy, cell apoptosis in I/R rats, or in H/R-induced SH-SY5Y cells. This suggests that there is an internal relationship between CI/R-induced inflammation and autophagy.

Recent studies demonstrated that TLR4 has an important role in mediating inflammation and autophagy in many CNS diseases, such as stroke and sclerosis [25]. Inhibition of TLR4 protects experimental stroke animals from injury [10, 11]. Recently, it is reported that TLR4-dependent autophagy is critical for macrophage-induced inflammation [25]. This result is consistent with our finding that TLR4 is upregulated in the CI/R injury rat or in the H/R-induced SH-SY5Y cells. This response is associated with increases in the expression levels of NLRP3 and LC3. These results demonstrate that CI/R-induced TLR4 upregulation is critical for activating inflammation and autophagy. This dependence is consistent with our finding showing that in the TLR4-deficient/knockout mice, an ischemic insult was associated with a smaller inflammatory response [10]. In addition, a previous study reported that TLR4-knockdown attenuates brain damage via inhibiting inflammation and autophagy in traumatic brain injury rats [26]. Considering the cause-and-effect relationship between ischemic stroke and traumatic brain injury, it is apparent that TLR4 is a potential therapeutic target for CI/R injury treatment.

However, considering the complexity of an interaction between inflammation and autophagy, there is no definitive mechanism that describes the role of TLR4 in CI/R injury. For example, the mechanism is unknown that accounts for how TLR4 regulates NLRP3 inflammasome and autophagy. It is still not clear if TLR4-mediated inflammation promotes autophagy or TLR4-mediated autophagy promotes inflammation in CI/R injury. Thus, much effort is still required to fully elucidate the role of TLR4 in inducing inflammation and autophagy in cerebral ischemia/reperfusion injury.

In summary, TLR4 plays an important role in inducing inflammation and autophagy in CI/R injury. We show here that brain CI/R injury induced-TLR4 activation promotes inflammation and autophagy via upregulating NLRP3 inflammasome expression and activity. This finding may lead to the development of novel approaches to improve therapeutic management of ischemic episodes in a clinical setting.

## Data Availability

The dataset can be available from the corresponding author if reasonably requested.
